# Supplementary material for: Challenges in access and satisfaction with reproductive, maternal, newborn and child health services in Nigeria during the COVID-19 pandemic: A cross-sectional survey
Source: PLoS One. 2021 May 7;16(5):e0251382. doi: 10.1371/journal.pone.0251382 (PMC8104439; doi:10.1371/journal.pone.0251382)
Supplement: S1 Questionnaire — (DOCX) [file pone.0251382.s001.docx]

**QUESTIONNAIRE TO ASSESS CLIENT’S SATISFACTION WITH ROUTINE RMNCH SERVICES DURING COVID-19 OUTBREAK IN LAGOS STATE**

**PERSONAL CHARACTERISTICS**

1. How old are you as at last birthday? _____ years
2. Marital status

a) Single

b) Married

c) Co-habiting

c) Separated

d) Divorced

e) Widowed

1. Religion

a) Christianity

b) Islam

c) Traditional

d) Others (specify) _________________________

1. Ethnicity

a) Yoruba

b) Igbo

c) Hausa

d) Others (specify) _________________________

1. Level of education

a) No formal education

b) Primary school uncompleted

c) Primary school completed

d) Secondary school uncompleted

e) Secondary school completed

f) Post-secondary (specify)_____________

g) Religious schooling only

h) Literacy classes only

1. Employment status
2. Employed
3. Unemployed
4. If employed, what is your occupation?

a) Senior professional e.g. Doctor, Lawyer, Accountant etc

b) Intermediate professional e.g. RN Nurses, Teachers, Secretaries etc

c) Junior professional/skilled e.g. Tailor, Typist, Auxiliary Nurses, Hairdressers,

Caterers etc

d) Semi-skilled e.g. Traders, Ward maids, School assistants, Food vendors, Nannies etc

e) Unskilled e.g. Cleaners, House helps, Labourers, Farmers etc

1. If unemployed, are you?
2. Housewife
3. Students
4. Apprentices
5. Retiree
6. Other:
7. In general, how would you rate your health today?
8. Very bad
9. Bad
10. Moderate
11. Good
12. Very good
13. What service(s) have you received in this facility since the COVID-19 outbreak in March till now? (multiple responses allowed)
    1. Antenatal care
    2. Delivery
    3. Postnatal care
    4. Family planning
    5. Post-abortion care
    6. PMTCT
    7. STI care
    8. Immunization
    9. Growth monitoring
    10. Treatment of childhood illness
    11. Newborn care
    12. Other (specify)……………………….
14. Other than this visit, when was your last visit to this facility to receive care for you or your child?_________________________
15. What kind of challenges have you had accessing health services at this facility since the COVID-19 outbreak in March? (multiple responses allowed)
    1. No challenge
    2. Couldn’t leave house because of lockdown
    3. No transport
    4. Facility closed
    5. Health workers not at facility
    6. Hostile health workers
    7. Other (specify)…………………………..

**CLIENTS SATISFACTION WITH MNCH SINCE ONSET OF** **COVID-19 OUTBREAK**

Kindly indicate your satisfaction with the services you have received since March

| Items | Not at all satisfactory | Somewhat satisfactory | Completely satisfactory | Not sure |
| --- | --- | --- | --- | --- |
| **Health care delivery** |  |  |  |  |
| Clinical examination |  |  |  |  |
| Diagnostic skills |  |  |  |  |
| Prescription of drugs |  |  |  |  |
| Quality of dispensed drugs |  |  |  |  |
| Recovery of patient |  |  |  |  |
| Monitoring of patient’s recovery |  |  |  |  |
| Fee for provided service |  |  |  |  |
| **Health Facility** |  |  |  |  |
| Adequacy of medical equipment |  |  |  |  |
| Adequacy of consulting rooms |  |  |  |  |
| Adequacy of staffing |  |  |  |  |
| Adequacy of health workers for women and children’s health |  |  |  |  |
| **Interpersonal aspects of care** |  |  |  |  |
| Compassion for patients |  |  |  |  |
| Respect for patients |  |  |  |  |
| Openness to patients |  |  |  |  |
| Honesty |  |  |  |  |
| Time spent to explain health status of the woman or child |  |  |  |  |
| Time devoted to patient |  |  |  |  |
| **Access to services** |  |  |  |  |
| Distance to commune to health centre |  |  |  |  |
| Ease of obtaining drugs |  |  |  |  |
